# Surveillance of Acute Respiratory Infections Using Community-Submitted Symptoms and Specimens for Molecular Diagnostic Testing

**DOI:** 10.1371/currents.outbreaks.0371243baa7f3810ba1279e30b96d3b6

**Published:** 2015-05-27

**Authors:** Jennifer Goff, Aaron Rowe, John S. Brownstein, Rumi Chunara

**Affiliations:** Boston University, Boston, Massachusetts, USA; San Francisco, California, USA; Boston Children’s Hospital, Harvard Medical School, Boston, Massachusetts, USA; New York University, New York, New York, USA; Boston Children's Hospital, Boston, Massachusetts, USA

**Keywords:** Influenza, participatory, Surveillance

## Abstract

Participatory systems for surveillance of acute respiratory infection give real-time information about infections circulating in the community, yet to-date are limited to self-reported syndromic information only and lacking methods of linking symptom reports to infection types. We developed the GoViral platform to evaluate whether a cohort of lay volunteers could, and would find it useful to, contribute self-reported symptoms online and to compare specimen types for self-collected diagnostic information of sufficient quality for respiratory infection surveillance. Volunteers were recruited, given a kit (collection materials and customized instructions), instructed to report their symptoms weekly, and when sick with cold or flu-like symptoms, requested to collect specimens (saliva and nasal swab). We compared specimen types for respiratory virus detection sensitivity (via polymerase-chain-reaction) and ease of collection. Participants were surveyed to determine receptivity to participating when sick, to receiving information on the type of pathogen causing their infection and types circulating near them. Between December 1 2013 and March 1 2014, 295 participants enrolled in the study and received a kit. Of those who reported symptoms, half (71) collected and sent specimens for analysis. Participants submitted kits on average 2.30 days (95 CI: 1.65 to 2.96) after symptoms began. We found good concordance between nasal and saliva specimens for multiple pathogens, with few discrepancies. Individuals report that saliva collection is easiest and report that receiving information about what pathogen they, and those near them, have is valued and can shape public health behaviors. Community-submitted specimens can be used for the detection of acute respiratory infection with individuals showing receptivity for participating and interest in a real-time picture of respiratory pathogens near them.

## Introduction

Internet connectivity has opened the door to new techniques for disease detection worldwide^1^. These ideas have been applied to influenza, given its annual occurrence and the potential for pandemics and emerging infections^2^. Accurate and rapid surveillance of influenza and other acute respiratory infections could: reduce the costs associated with unnecessary investigations, facilitate more timely (and hence more effective) use of antiviral drugs for influenza, help prevent the secondary spread of infection, reduce the unnecessary use of antibiotics and shorten hospital stays^3^. Over the last decade, Internet-based influenza surveillance systems have been developed and deployed nationally in Australia, the United States and Canada and 10 countries in Europe^4^^,^ 5 ^,^ 6. These systems have pioneered the ability to involve individual volunteers in public health through a participatory process; influenza and acute respiratory illness-related symptoms are collected from community members who voluntarily report their health status on a weekly basis via web and mobile apps. Data from these systems have demonstrated the potential to better understand acute respiratory illness in the population by detecting trends early and at the point of contact, piecing together real-time patterns of disease spread, identifying important subpopulations, monitoring the course of an epidemic or pandemic, and helping to target prevention and control efforts. However, none of these Internet-based systems employ molecular diagnostics to rapidly confirm the viral cause of illness, which is important because the clinical presentation of influenza is similar to illness caused by other respiratory pathogens^7^.

New detection technologies are rapidly emerging for home-based and point of care diagnosis; they harness real-time polymerase chain reaction as well as newer techniques for high sensitivity and specificity molecular detection^8^^,^ 9. In all of these technologies, there are common threads needing investigation, with respect to the types of specimens, both in regards to feasibility of viral detection as well as the willingness and capability of individuals to generate the sample on their own. Specimens for molecular detection of acute respiratory infections generally consist of nasopharyngeal wash and aspirate as the gold standard, however there are safety concerns for lay volunteers to collect this type of specimen at home^10^. Nasal swabs have been successfully used by volunteers to collect viable samples for influenza culture at home, at first for individuals whom were classified by nurses to have 'colds and flu' 11 ^,^ 27. Further, nasal self sampling has been used as part of population-based surveillance of respiratory virus infections and compared to contemporaneous clinical surveillance specimens and general practitioner sampling in some European cohorts 26 ^,^ 28. To date, none of these systems have considered other specimen types, or reported how individuals' symptoms were linked to detected infection.

While nasal swabs are easy to use, the non-invasive nature of oral specimen collection makes it an easier and more convenient and thus preferable alternative to nasal swabs^15^^,^ 16. Oral specimens have been widely used to obtain genetic material for direct-to-consumer medical tests^14^. As well, although the literature is sparse, oral specimens have been shown to be reliable in the detection of certain respiratory pathogens. Saliva were found to be accurate and reliable in the detection of H1N1 and comparable to the nasopharyngeal swab^18^. Oral specimens have also been found to be effective in the detection of SARS, measles and beta-hemolytic streptococcus in pediatric patients^12^^,^ 13 ^,^ 15 ^,^ 16 ^,^ 17. Others have found oral specimens to be inferior to nasal swabs in the detection of respiratory viruses, but still suitable in settings where obtaining a nasal swab would be impractical^21^. Beyond specimen type, community participation in analysis and specimen collection for flu surveillance is also unexplored. Academic and commercial entities have demonstrated^19^^,^ 20 ^,^ 21 that home sample collection is feasible from the community and at scale for genomic, microbiome and STD testing^23^^,^ 24, however the utility and format of what information should be communicated back to individuals, in order to be useful to impact public health behaviors is a related and open question.

In sum, while there have been some efforts to use nasal swabs for self-collection of specimens and surveillance of acute respiratory infection at scale, the work reported here adds to these existing efforts by considering important questions of utility of different specimen types, linking of individual participatory symptom reports to specimen types, and how the resulting surveillance information can be used at an individual level. To address these needs, we developed the GoViral platform. This platform is designed to acquire community generated diagnostic samples and couple them with other contemporaneous information such as participatory syndromic reports. Thus, GoViral enables us to test: 1) the types of samples that individuals are able to and willing to generate in their own homes in a timely fashion when they have an acute respiratory infection along with and appropriate processing methods, 2) how user-generated diagnostic information corresponds to self-reported symptoms and 3) reception and use of the information by the public. Herein we report results from an initial deployment during the 2013-2014 influenza season

## Materials and Methods

**Participant Recruitment and Sign Up**

A cohort of lay volunteers was recruited in Massachusetts. Recruitment was open throughout the study period (November 1, 2013 to April 30, 2014). We used a convenience sample in order to rapidly recruit participants and evaluate the platform during this first season (thus anticipating participants to be skewed towards those who have access to Internet), permitting investigation via a more representative sampling during subsequent years. Enrollment was driven largely by announcements on local radio stations and word of mouth. Paid online advertisements and social media were also used as means of recruiting volunteers to the study. The study size was limited by our ability to recruit and engage participants. In order to register, volunteers signed an electronic consent form and reported their email address, name and mailing address, gender and age. Volunteers were immediately sent a kit (see section below) to keep at home. Users were instructed that if they became sick with symptoms of a cold or flu, they should perform the specimen collection and rapid test within 48 hours of experiencing symptoms. Participants who returned a kit were sent a replacement, allowing them to report a second illness (second illness was included if the specimen was provided at least 2 weeks after the initial specimen). GoViral participants were also registered for Flu Near You and reported symptoms through weekly surveys, as has been previously described^6^. If a participant reported any symptoms on their weekly survey, they immediately were sent an email reminder to follow the instructions in the kit and submit specimens. Instructions for submission of the kit were made available on a report page. On the report page, an individual confirms the symptoms that they have, can view video instructions (Supplementary Information) for each of the sample types and rapid test, and they record the date illness began, the numbering on their sample tubes, and test results from the rapid test. All participants who signed up with a Massachusetts mailing address were sent a kit and their symptom reports were included in the study.

**Ethics**

The protocol was approved by the Boston Children’s Hospital Institutional Review Board.

**Kit Contents**

Each kit (Figure 1) included an Oragene OM-505 Saliva collection tube (DNA Genotek Inc.), flocked nasal swab with viral collection media (part number 407C, Diagnostic Hybrids), and a lateral flow immunoassay rapid test (CerTest Biotec). The CerTest rapid test tested for Influenza A, Influenza B, RSV, and adenovirus. For the nasal specimen, we selected a nasal swab rather than a nasopharyngeal swab because it is easier to collect for the individual, and a previous study has demonstrated that they are comparable in sensitivity when using molecular diagnostic tests^13^. Self-collected flocked swabs have also been found to be equivalent in cell yield compared to staff collected flocked swabs^22^ and respiratory cell yields were higher when using flocked nasal swabs compared to rayon swabs^14^. For the saliva sample, we used the Oragene Saliva collection tube which has been designed for the collection and stabilization of microbial DNA and RNA from saliva. Samples are reported to remain stable at room temperature for up to 3 weeks in the stability solution included in the tube^23^. The kits, along with prepaid postage were mailed to participants at the beginning of the influenza season with pre-paid return overnight postage. We used USPS so that participants simply had to return the kits by dropping them off in any mailbox or post office. Kits were paired with customized written instructions and instructional videos (see specimen collection section).

**Photo of specimen collection kit with components. Each of the kits sent to participants included a a) rapid test, b) rapid test liquid reagent, c) nasal swab tube with transport medium, d) oral Specimen collector and e) flocked nasal swabs.**
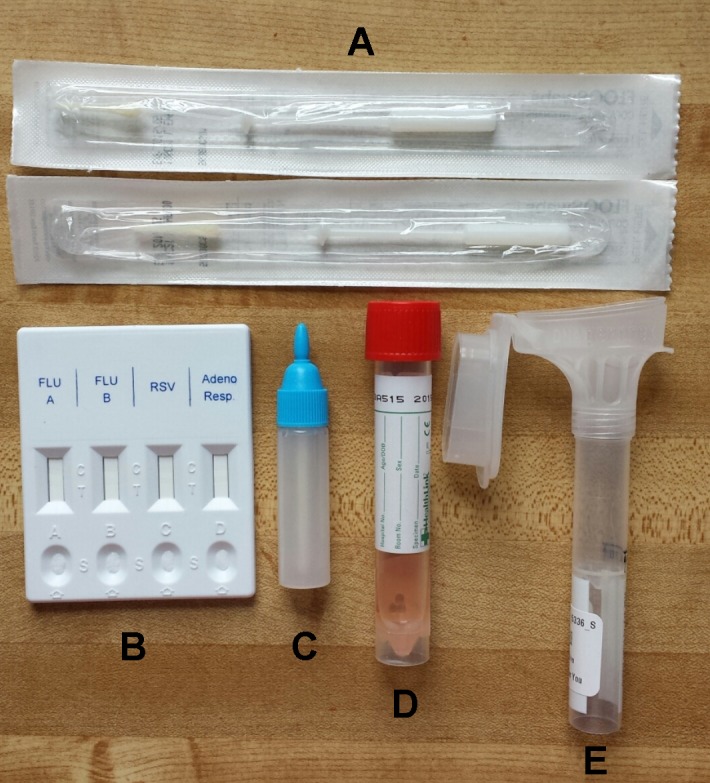

**Specimen Collection**

Participants were instructed to collect a nasal sample by inserting the flocked nasal swab approximately 2.5 cm into one nostril and rotating. The swab was then placed in transport media for testing. 2 mL of saliva in transport media was collected. A second nasal swab mixed with reagent was collected and then the reagent was used as per instructions for the rapid test 27. From the written GoViral kit instructions participants were directed to the GoViral website to submit symptom information, kit number, and photo of rapid test results. Customized YouTube instructional videos (https://www.youtube.com/watch?v=GWRMbvFjRsg, https://www.youtube.com/watch?v=jAQ-OF8-cyA, https://www.youtube.com/watch?v=S1oE4DLDiw8) showed participants how to collect the oral and nasal specimens and package them for mailing. Samples were sent by overnight mail to the laboratory for detection.

**Specimen Preparation and Analysis**

Specimens received were excluded (both the pair of nasal and saliva specimens) if they were received and had leaked due to poor packaging or appeared to not have any saliva added. We tested samples on both the GenMark Respiratory Viral Panel, and Biofire FilmArray Respiratory Panel. Both offer a multiplex panel for respiratory pathogens. They however differ in the DNA/RNA extraction methods used, in the measured sensitivity and specificity (for nasopharyngeal specimens), and workflow parameters, such as hands-on time, time to result, and relative ease of use. Previous studies have compared these molecular multiplex platforms including laboratory developed molecular methods (PCR)^25^. While all of these studies were using nasopharyngeal (NP) swab specimens, because of the different nucleic extraction methods in each platform and potential differences in viral load and location in saliva versus nasal specimens, their performance on these sample types is still indeterminate.

**Detection on Genmark**

Prior to detection on the Genmark assay, DNA/RNA were extracted from the samples using the MagNA Pure extraction protocol. Details of this and further procedure are reported in the Supplementary Information section. The Genmark Panel includes: Influenza A (Subtype to Influenza A H1, Influenza A H3 and Influenza A 2009 H1N), Influenza B, Respiratory Syncytial Virus (RSV) A, Respiratory Syncytial Virus (RSV) B, Parainfluenza Virus (PIV) 1, Parainfluenza Virus (PIV) 2, Parainfluenza Virus (PIV) 3, Human Metapneumovirus (hMPV), Human Rhinovirus (HRV), Adenovirus B/E and Adenovirus C. Viral targets in the FilmArray are Influenza A (Influenza A H1, Influenza A H3, Influenza A 2009 H1N1), Influenza B, Respiratory Syncytial Virus (RSV), Coronavirus HKU1, Coronavirus NL63, Coronavirus 229E, Coronavirus OC43, Parainfluenza Virus (PIV) 1, Parainfluenza Virus (PIV) 2, Parainfluenza Virus (PIV) 3, Parainfluenza Virus (PIV) 4, Human Metapneumovirus (hMPV), Human Rhinovirus/Enterovirus and Adenovirus. The result is not binary, a threshold cycle (*C­­_t _*) of 3.0, determined through experience with the Genmark system, is used to classify a pathogen as detected or not in our analysis.

**Detection on BioFire FilmArray**

Specimens were processed following the procedure found in the FilmArray Respiratory Panel Instruction Booklet. The FilmArray RP contains its own extraction procedure and internal controls within each pouch. In the FilmArray, DNA/RNA is extracted from the sample through physical agitation and chemical disruption of the cells with beads. Possible results include Detected, Not Detected, Equivocal (Influenza A only) and Invalid.

**Survey**

In order to gauge if diagnostic test results or flu risk actually influence individuals to change behaviors, participants were sent a survey at the completion of the study period. One of two versions of the survey was sent depending on whether the participant had sent in diagnostic samples. Participants were asked about their preventative behaviors and the difficulty of the specimen collection protocols (all questions and responses in Table S1).

**Outcomes and Statistical Methods**

Analyses were performed using R version 2.15.1 (R Development Core Team, Vienna, Austria). Key outcomes of interest were self-reported symptoms (participants could report any of fever, cough, sore throat, shortness of breath, chills, fatigue, nausea, diarrhea, body aches and headache), and diagnostic results for each of the nasal and saliva specimens via both the Genmark and BioFire systems for each set of specimens sent in. Timing of specimen collection compared to symptom onset was noted in order to understand the potential of self-collection. Univariate comparisons between symptoms and diagnostic results were performed, but sample size was too low to warrant further analyses.

## Results

**Participant Demographics and Participation**

Between November 1, 2013 and April 30, 2014, 295 participants enrolled in the study and received a kit. During the study period we also sent a second kit to participants who already completed one kit, allowing for second illnesses to be captured. Among the cohort of enrolled study participants, 167 individuals reported any symptoms during the study time frame, and from those individuals 71 pairs of diagnostic samples were returned (1 returned nasal sample had no accompanying saliva sample), and sent for nucleic acid analysis. Participants submitted kits an average of 3.29 (95% CI: 2.39, 4.18) days after symptom onset (Figure 2). People ages 20 to 77 signed up and received kits (Figure 3), and 70% of signups were women. Kits were returned by individuals ranging in age from 20 to 70.****

**Days between symptom onset and specimen submission. The length of time was computed based on self-report of illness onset date by the participant and the date they completed a specimen submission form.**
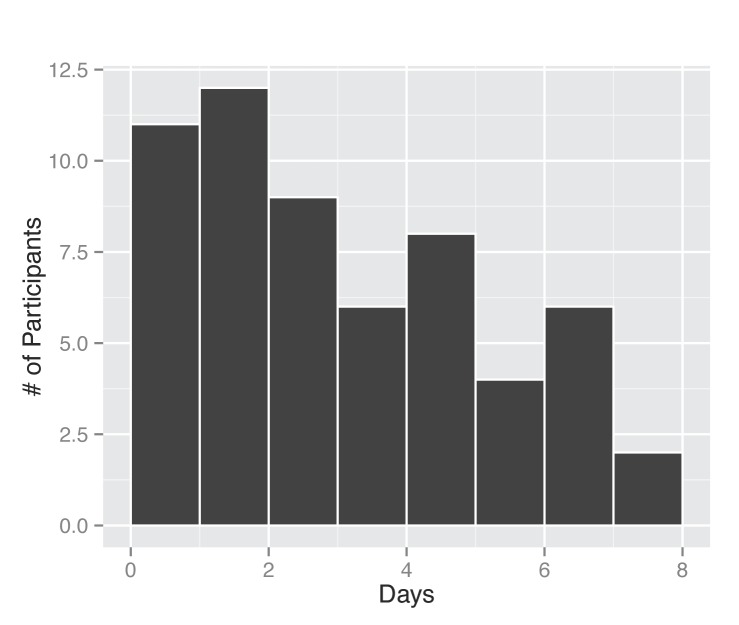

**Gender and ages of participants. Demographics of all individuals who signed up as participants, those who ended up returning a kit or did not.**
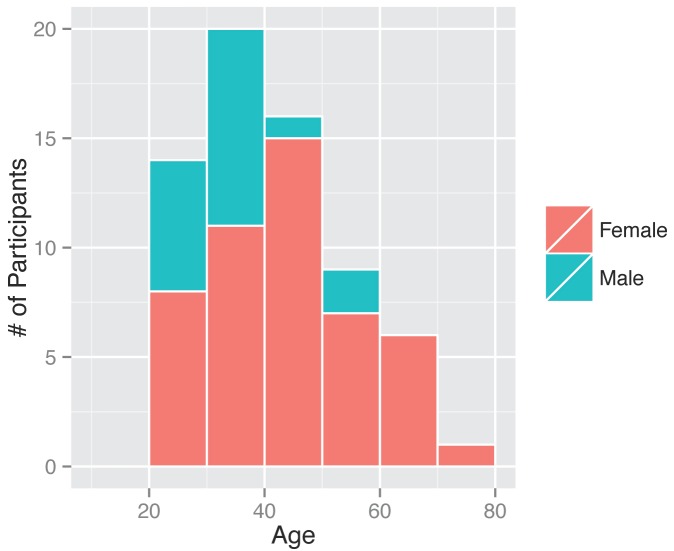

**Pathogens Detected (BioFire FilmArray, Genmark and rapid tests)**

Out of the 71 pairs of samples tested, 35 sets of samples tested positive in at least one specimen on both systems, 31 were negative on both and there were 5 cases in which both the saliva and nasal specimens tested on one system did not detect a virus detected in at least one specimen using the other system: Influenza A was detected in one nasal specimen by Genmark (and not on the FilmArray), Coronavirus was detected in one saliva sample detected by Genmark (and not on the FilmArray), Coronavirus detected in one nasal sample by FilmArray (and not on the Genmark), Coronavirus detected in one saliva sample by FilmArray (and not the Genmark), RSV was detected in both a nasal and saliva samples from one person on the FilmArray (and not on the Genmark). The distribution of samples and viruses detected in at least one specimen (saliva or nasal) on both systems are illustrated in Figures 4a and b. In 10 cases, there was a difference between results for the nasal and saliva specimen pairs on at least one of the systems, but the same pathogen was also detected in the other system (Table S2).

**Aggregate results by pathogen. Overall results, as a proportion of all participants that had a positive result in either specimen on both systems, for the a) Genmark and b) BioFire FilmArray.**
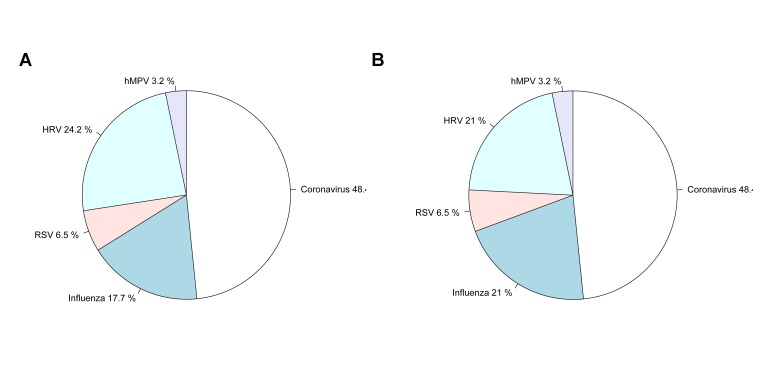

Rapid immunoassays performed by the users were not useful in identifying the specimens that were positive with influenza, RSV or adenovirus. None of the rapid tests returned alongside any of the 71 returned pairs of specimens demonstrated positive results for any pathogens, even ones that were determined positive by the PCR-based methods.

**Relationship Between Symptoms and Diagnostic Results**

Of people who reported The CDC (Centers for Disease Control and Prevention)’s “influenza-like illness” (ILI) symptom profile (fever and cough or sore throat) two out of twelve of tested positive for influenza, five out of twelve did not test positive for anything on the panel and five out of twelve tested positive for other viruses (Coronavirus, hMPV and RSV). The full summary of symptom profiles by pathogen is provided in Table S3.

**Participant Survey Results**

Results of the survey are reported in Table S1. Overall, it was found that i) most participants who sent in a kit did not seek any interventions from healthcare professionals for their illness (86.7%), ii) it was harder for people to produce the nasal sample than the saliva sample, and iii) beyond finding out their own diagnosis, most individuals reported that they would find it also useful to know what others in their area have had near them (83.3% for those who sent in kits, 93.7% for those who did not).

## Discussion

Our study seeks to specifically study user preferences and specimen types towards building a scalable method connecting self-reported symptoms to infection type for understanding community prevalence of respiratory infections. Multiple initial findings are offered: diagnostic samples can be generated by the public demonstrated by the high user participation rate (half of the people who reported any symptoms at all completed the kit and sent in specimens), and initial validation of oral specimens as compared with nasal specimens can be used for the identification of upper respiratory pathogens, along with reports of greater comfort and preference for oral specimens. While we anticipate the participation rate is biased upwards based on the types of people who may have agreed to participate in the study, it indicates that while sick at home, people are willing and able to complete the specimen collection. Influenza A, RSV, Coronavirus (3 subtypes), hMPV and HRV were detected from saliva and nasal samples, with high concordance between the nasal and saliva samples measured on two different detection methods. Pairing symptom profiles with the detected viruses showed that most people who self-report the common ILI profile did not have influenza. Through our survey, we found that most individuals who became ill and returned a sample did not seek healthcare, such as going to a clinic or the hospital. Overall, saliva collection was reported to be easier than the nasal swab specimen collection. Rapid immunoassays did not show value for community use, compared to the PCR detection of community-acquired specimens. Beyond the expected lower sensitivity and specificity of the rapid tests, the specifications for these tests have been determined using nasopharyngeal swabs on children, thus the performance on specimens from adults and using nasal swabs can be attributed to this difference. The different assays, GenMark and BioFire FilmArray, demonstrated similar performance with some false negatives on both saliva and nasal specimens from each system as compared to the other. We also found that individuals would find it useful to know what pathogens were detected in other people in the community. Regarding finding out their own diagnosis, most common reasons cited for desiring this information included concern for exposure to others, especially by those who also mentioned they work in healthcare. The results presented here suggest methods for achieving these preferences, and with further studies involving more and diverse groups the power of these conclusions will increase.

There are many aspects of the study which can be improved and future research should address. One limitation of this study was the small and non-representative sample of volunteers. Based on the Internet or mobile requirement for participation, as well as a self-selective nature of interest, participants of this study are similar demographically to those in other participatory systems^6^. Geographically, recruitment was purposefully limited but will be expanded in the future to target under-represented groups. Although the number of samples received was consistent with the expected number of individuals who would be sick with some type of respiratory infection, this indicates that in order to increase the number of samples, a greater number of participants are required. Finally, while rapid tests were included in the kits and obtaining rapid results is an incentive for individuals to participate, the performance was not specific nor sensitive enough to warrant further use. Different rapid diagnostic tests may be experimented with in forthcoming kits, as while the majority of individuals report they would like to receive the results of their own tests within two days, which is feasible with this model, a more real-time test is optimal for decision making.

This study represents an advance with respect to the field of participatory public health surveillance systems. To-date, reports in the literature of online participatory studies have been limited to self-reported syndromic information only. Some studies have combined self-reported influenza symptoms with serology to understand attack rates in the population. However, linkage to symptoms has been limited in self-sampling studies, and although other studies have demonstrated that individuals can obtain a nasal swab specimen from themselves, we report here that individuals prefer saliva and initial results indicate that it can potentially provide a feasible medium for acute upper respiratory infection detection. Finally, via the results of our survey, it is indicated that participatory information (crowdsourced) can be used to generate context-specific, rapid information for disease surveillance that has the potential to impact public health behaviors.

Going forward, further study of data from the GoViral surveillance method offers potential benefits such as understanding household transmission characteristics, enabling evaluation of the relationship between reported symptom profiles and viral etiology as well as spatio-temporal distribution of upper respiratory infection, duration of symptoms and its relation to viral etiology, and impact of these efforts on an individual’s public health and healthcare seeking behavior. Beyond a cohort approach here, community specimen collection can be used during seasonal outbreak or pandemic investigations. Future work will increase the number and representatively of study participant demographics, as well as employ next-generation detection technologies to improve sensitivity and speed of generating the diagnostic results.

## Competing Interests

All authors have confirmed that they have no financial, personal or professional competing interests to report.

## Table S1. All specimen results on both Biofire and Genmark

**1. Do you take any preventative measures during flu season (multiple choices allowed)?**

Sent in kits (n = 29 responses)

**Table:** 

Work/studying from home	3 (10%)
Extra hand washing	18 (62%)
Got a vaccine	19 (65%)
Avoiding contact with others	8 (28%)
Didn’t take any	2 (7%)

Did not send in kits (143 responses)

**Table:** 

Work/studying from home	14 (10%)
Extra hand washing	63 (44%)
Got a vaccine	106 (74%)
Avoiding contact with others	20 (14%)
Didn’t take any	21 (15%)

**2. Did your illness this season for which you sent in a sample prevent you (or the household member who’s specimen it was) from participating in any normal activities (work, school) If yes, for how many days?**

Sent in kits (28 responses)

**Table:** 

No	12 (43%)
1-2 days	10 (36%)
3 days	3 (11%)
1 week	1 (4%)
10 days	1 (4%)
Can’t remember	1 (4%)

**3. Did you go see a doctor, or go into the hospital or seek some kind of care because of your illness?**

Sent in kits (30 responses)

**Table:** 

Yes	4 (13%)
No	26 (87%)

**4. What part of the process of submitting your sample was easiest?**

Sent in kits (29 responses)

**Table:** 

Filling out the online form	20 (69%)
Using nasal swab	4 (14%)
Saliva collection	5 (17%)

**5. What part of the process of submitting your sample was hardest?**

Sent in kits (26 responses)

**Table:** 

Filling out the online form	5 (20%)
Using nasal swab	12 (46%)
Saliva collection	9 (35%)

**6. Would you find it useful to know the result of what you had when start feeling sick?**

Sent in kits (30 responses)

**Table:** 

Within 1 day	11 (37%)
Within 2 days	9 (30%)
Within a week	4 (13%)
Just finding out at end of season is fine	6 (20%)

Did not send in kit (138 responses)

**Table:** 

Within 1 day	34 (25%)
Within 2 days	33 (24%)
Within a week	26 (19%)
Just finding out at end of season is fine	45 (33%)

**7. Would you find it useful to know what others in your area have had near you (such as in the map?)**

Sent in kits (30 responses)

**Table:** 

Yes	25 (83%)
No	5 (17%)

Did not send in kit (141 responses)

**Table:** 

Yes	132 (94%)
No	9 (6%)

**8. What would you do with this information?**

Sent in kits (27 responses)

**Table:** 

Just for information/curiosity	6/27 (22%)
Take appropriate precautions for self/others	11/27 (41%)
Tell doctor	3/27 (11%)
Doctor and activities	5/27 (19%)
Nothing	2/27 (7%)

Didn’t send in a kit (110 responses)

**Table:** 

Just for information/curiosity	34/110 (31%)
Take appropriate precautions for self/others	51/110 (46%)
Tell doctor	14/110 (13%)
Doctor and activities	3/110 (3%)
Nothing	8/110 (7%)

## Table S2. All specimen results on both the Biofire FilmArray and Genmark

**Table:** 

Sample Type	Genmark Result	FilmArray Result
Nasal	Infl A 2009 H1N1 Ct=136.2Infl A Ct=115	none
Saliva	Infl A 2009 H1N1 Ct=137.3	FLA
Nasal	negative	none
Saliva	negative	none
Nasal	Corona 229E Ct=226.3	CORONA
Saliva	Corona 229E Ct=141.4	CORONA
Nasal	negative	none
Saliva	negative	none
Nasal	negative	none
Saliva	negative	none
Nasal	Corona 229E Ct=264.2	CORONA
Saliva	Corona 229E Ct=235.7	CORONA
Nasal	Infl A 2009 H1N1 Ct=347Infl A Ct=201.3	FLA
Saliva	negative	none
Nasal	negative	none
Saliva	negative	none
Nasal	Corona HKU1 Ct=243.3	CORONA
Saliva	Corona HKU1 Ct=279	CORONA
Nasal	negative	none
Saliva	negative	none
Nasal	negative	EN-RH
Saliva	negative	EN-RH
Nasal	Corona NL63 Ct=6.9	CORONA
Saliva	Corona NL63 Ct=16.5	CORONA
Nasal	Infl A 2009 H1N1 Ct=348Infl A Ct=155	FLA
Saliva	Infl A 2009 H1N1 Ct=80.2Infl A Ct=208	FLA
Nasal	Infl A 2009 H1N1 Ct=210Infl A Ct=225	FLA
Saliva	negative	none
Nasal	Infl A 2009 H1N1 Ct=160Infl A Ct=97.6	none
Saliva	negative	FLA
Nasal	Corona 229E Ct=9.1	none
Saliva	Corona 229E Ct=5.8	CORONA
Nasal	negative	none
Saliva	negative	none
Nasal	Corona HKU1 Ct=269	CORONA
Saliva	Corona HKU1 Ct=240	CORONA
Nasal	negative	none
Saliva	negative	none
Nasal	RSV A Ct=176	RSVI
Saliva	RSV A Ct=152	RSVI
Nasal	negative	none
Saliva	negative	CORONA
Nasal	Corona 229E Ct=202Corona HKU1 Ct=279	CORONA
Saliva	Corona 229E Ct=4.3Corona HKU1 Ct=169	CORONA
Nasal	negative	none
Saliva	negative	none
Nasal	Corona 229E Ct=197.2	CORONA
Saliva	Corona 229E Ct=206.5	CORONA
Nasal	negative	none
Saliva	negative	none
Nasal	negative	none
Saliva	negative	none
Nasal	negative	none
Saliva	negative	none
Nasal	negative	none
Saliva	negative	none
Nasal	Corona 229E Ct=206	CORONA
Saliva	Corona 229E Ct=207	CORONA
Nasal	Corona HKU1 Ct=184	none
Saliva	negative	CORONA
Nasal	negative	none
Saliva	negative	none
Nasal	Corona HKU1 Ct=153	CORONA
Saliva	negative	none
Nasal	negative	none
Saliva	negative	none
Nasal	negative	none
Saliva	negative	none
Nasal	Corona HKU1 Ct=213	CORONA
Saliva	negative	none
Nasal	negative	none
Saliva	negative	none
Nasal	negative	none
Saliva	negative	none
Nasal	negative	none
Saliva	negative	none
Nasal	negative	none
Saliva	negative	none
Nasal	CORONA NL63 Ct=5.3	CORONA
Saliva	negative	CORONA
Nasal	negative	CORONA
Saliva	negative	none
Nasal	negative	none
Saliva	negative	none
Nasal	negative	none
Saliva	negative	none
Nasal	negative	none
Saliva	negative	none
Nasal	negative	none
Saliva	negative	none
Nasal	negative	none
Saliva	negative	none
Nasal	Infl A 2009 H1N1 Ct=229Infl A Ct=233	FLA
Saliva	Infl A 2009 H1N1 Ct=312Infl A Ct=253	FLA
Nasal	negative	none
Saliva	negative	none
Nasal	Corona HKU1 Ct=3.8	none
Saliva	Corona HKU1 Ct=91.4	CORONA
Nasal	negative	none
Saliva	negative	none
Nasal	Corona 229E Ct=105	none
Saliva	Corona 229E Ct=197	CORONA
Nasal	negative	none
Saliva	negative	none
Nasal	negative	none
Saliva	HRV Ct=180	EN-RH
Nasal	negative	none
Saliva	Corona 229E Ct=5.8	none
Nasal	negative	none
Saliva	negative	none
Nasal	negative	none
Saliva	negative	none
Nasal	RSV A Ct=201	RSVI
Saliva	RSV A Ct=137	RSVI
Nasal	Corona NL63 Ct=16.5	CORONA
Saliva	negative	CORONA
Nasal	HRV Ct=17.1	EN-RH
Saliva	HRV Ct=25.4	EN-RH
Nasal	negative	none
Saliva	negative	none
Nasal	Infl A H3 Ct=148 Infl A Ct=32.4	FLA
Saliva	Infl A H3 Ct=161 Infl A Ct=46.1	FLA
Nasal	negative	none
Saliva	negative	none
Nasal	Corona 229E Ct=167	CORONA
Saliva	Corona 229E Ct=48	CORONA
Nasal	hMPV Ct=140	HMPV
Saliva	hMPV Ct=120	HMPV
Nasal	HRV Ct=14.6	EN-RH
Saliva	HRV Ct=9.6	EN_RH
Nasal	Infl A H3 Ct=81.6 Infl A Ct=12	none
Saliva	negative	none
Nasal	Infl A H3 Ct=147 Infl A Ct=67.6	FLA
Saliva	negative	none
Nasal	HRV Ct=157	EN-RH
Saliva	HRV Ct=7	EN-RH
Nasal	HRV Ct=212	EN-RH
Saliva	HRV Ct=148	EN-RH
Nasal	negative	none
Nasal	HRV Ct=141	EN-RH
Saliva	HRV Ct=128	EN-RH
Nasal	HRV Ct=26.7	EN-RH
Saliva	HRV Ct=17.3	EN-RH

## Table S3: Proportion (%) of diagnosis with specific symptom profiles reported for each detected pathogen (total detected or reported (n)).

**Table:** 

Symptoms Reported	Influenza A (8)	Coronavirus (17)	HMPV (1)	HRV (7)	RSV (2)	Negative (31)
fever only (1)	0.0	0.00	0.00	0.0	0.0	3.2
cough only (7)	25.0	5.9	0.00	28.6	0.0	6.5
sore throat only (16)	12.5	41.2	0.00	28.6	0.0	19.4
none (19)	0.0	23.5	0.00	14.3	0.0	45.2
cough and sore throat (11)	37.5	11.8	0.00	28.6	50.0	9.7
fever, cough and sore throat (4)	0.0	5.9	100.0	0.00	0.0	6.5
fever and cough (4)	25.0	0.00	0.00	0.00	50.0	3.2
fever and sorethroat (4)	0.00	11.8	0.00	0.00	0.0	6.5

## References

[1] Brownstein JS, Freifeld CC, Madoff LC. Digital disease detection--harnessing the Web for public health surveillance. N Engl J Med. 2009 May 21;360(21):2153-5, 2157. PubMed PMID:19423867. 19423867 10.1056/NEJMp0900702 PMC2917042

[2] Neumann G, Noda T, Kawaoka Y. Emergence and pandemic potential of swine-origin H1N1 influenza virus. Nature. 2009 Jun 18;459(7249):931-9. PubMed PMID:19525932. 19525932 10.1038/nature08157 PMC2873852

[3] Byington CL, Castillo H, Gerber K, Daly JA, Brimley LA, Adams S, Christenson JC, Pavia AT. The effect of rapid respiratory viral diagnostic testing on antibiotic use in a children's hospital. Arch Pediatr Adolesc Med. 2002 Dec;156(12):1230-4. PubMed PMID:12444835. 12444835 10.1001/archpedi.156.12.1230

[4] van Noort SP, Muehlen M, Rebelo de Andrade H, Koppeschaar C, Lima Lourenço JM, Gomes MG. Gripenet: an internet-based system to monitor influenza-like illness uniformly across Europe. Euro Surveill. 2007 Jul 1;12(7):E5-6. PubMed PMID:17991409. 17991409 10.2807/esm.12.07.00722-en

[5] Carlson SJ, Dalton CB, Tuyl FA, Durrheim DN, Fejsa J, Muscatello DJ, Francis JL, d'Espaignet ET. Flutracking surveillance: comparing 2007 New South Wales results with laboratory confirmed influenza notifications. Commun Dis Intell Q Rep. 2009 Sep;33(3):323-7. PubMed PMID:20043603. 20043603 10.33321/cdi.2009.33.35

[6] Chunara R, Aman S, Smolinski M, Brownstein JS (2013) Flu near you: an online self-reported influenza surveillance system in the USA. Online Journal of Public Health Informatics 5.

[7] Cowling BJ, Chan KH, Fang VJ, Cheng CK, Fung RO, Wai W, Sin J, Seto WH, Yung R, Chu DW, Chiu BC, Lee PW, Chiu MC, Lee HC, Uyeki TM, Houck PM, Peiris JS, Leung GM. Facemasks and hand hygiene to prevent influenza transmission in households: a cluster randomized trial. Ann Intern Med. 2009 Oct 6;151(7):437-46. PubMed PMID:19652172. 19652172 10.7326/0003-4819-151-7-200910060-00142

[8] Lam B, Fang Z, Sargent EH, Kelley SO. Polymerase chain reaction-free, sample-to-answer bacterial detection in 30 minutes with integrated cell lysis. Anal Chem. 2012 Jan 3;84(1):21-5. PubMed PMID:22142422. 22142422 10.1021/ac202599b

[9] Das J, Cederquist KB, Zaragoza AA, Lee PE, Sargent EH, Kelley SO. An ultrasensitive universal detector based on neutralizer displacement. Nat Chem. 2012 Jun 3;4(8):642-8. PubMed PMID:22824896. 22824896 10.1038/nchem.1367

[10] Irving SA, Vandermause MF, Shay DK, Belongia EA. Comparison of nasal and nasopharyngeal swabs for influenza detection in adults. Clin Med Res. 2012 Nov;10(4):215-8. PubMed PMID:22723469. 22723469 10.3121/cmr.2012.1084 PMC3494547

[11] Daley P, Castriciano S, Chernesky M, Smieja M. Comparison of flocked and rayon swabs for collection of respiratory epithelial cells from uninfected volunteers and symptomatic patients. J Clin Microbiol. 2006 Jun;44(6):2265-7. PubMed PMID:16757636. 16757636 10.1128/JCM.02055-05 PMC1489401

[12] Karabay O, Ekerbicer H, Yilmaz F. Efficacy of throat gargling for detection of group a beta-hemolytic streptococcus. Jpn J Infect Dis. 2005 Feb;58(1):39-40. PubMed PMID:15728991. 15728991

[13] Hutse V, Van Hecke K, De Bruyn R, Samu O, Lernout T, Muyembe JJ, Brochier B. Oral fluid for the serological and molecular diagnosis of measles. Int J Infect Dis. 2010 Nov;14(11):e991-7. PubMed PMID:20851015. 20851015 10.1016/j.ijid.2010.06.009

[14] Tung JY, Do CB, Hinds DA, Kiefer AK, Macpherson JM, Chowdry AB, Francke U, Naughton BT, Mountain JL, Wojcicki A, Eriksson N. Efficient replication of over 180 genetic associations with self-reported medical data. PLoS One. 2011;6(8):e23473. PubMed PMID:21858135. 21858135 10.1371/journal.pone.0023473 PMC3157390

[15] Bilder L, Machtei EE, Shenhar Y, Kra-Oz Z, Basis F. Salivary detection of H1N1 virus: a clinical feasibility investigation. J Dent Res. 2011 Sep;90(9):1136-9. PubMed PMID:21700809. 21700809 10.1177/0022034511413283

[16] Karabay O, Ekerbicer H, Yilmaz F. Efficacy of throat gargling for detection of group a beta-hemolytic streptococcus. Jpn J Infect Dis. 2005 Feb;58(1):39-40. PubMed PMID:15728991. 15728991

[17] Wang WK, Chen SY, Liu IJ, Chen YC, Chen HL, Yang CF, Chen PJ, Yeh SH, Kao CL, Huang LM, Hsueh PR, Wang JT, Sheng WH, Fang CT, Hung CC, Hsieh SM, Su CP, Chiang WC, Yang JY, Lin JH, Hsieh SC, Hu HP, Chiang YP, Wang JT, Yang PC, Chang SC. Detection of SARS-associated coronavirus in throat wash and saliva in early diagnosis. Emerg Infect Dis. 2004 Jul;10(7):1213-9. PubMed PMID:15324540. 15324540 10.3201/eid1007.031113 PMC3323313

[18] Robinson JL, Lee BE, Kothapalli S, Craig WR, Fox JD. Use of throat swab or saliva specimens for detection of respiratory viruses in children. Clin Infect Dis. 2008 Apr 1;46(7):e61-4. PubMed PMID:18444806. 18444806 10.1086/529386 PMC7107872

[19] Turnbaugh PJ, Ley RE, Hamady M, Fraser-Liggett CM, Knight R, et al. (2007) The human microbiome project. Nature 449: 804-810 10.1038/nature06244 PMC3709439 17943116

[20] Eriksson N, Macpherson JM, Tung JY, Hon LS, Naughton B, et al. (2010) Web-based, participant-driven studies yield novel genetic associations for common traits. PLoS genetics 6: e1000993. 10.1371/journal.pgen.1000993 PMC2891811 20585627

[21] Kløvstad H, Natås O, Tverdal A, Aavitsland P (2013) Systematic screening with information and home sampling for genital Chlamydia trachomatis infections in young men and women in Norway: a randomized controlled trial. BMC infectious diseases 13: 30. 10.1186/1471-2334-13-30 PMC3558461 23343391

[22] Smieja M, et al. (2010) Development and Evaluation of a Flocked Nasal Midturbinate Swab for Self-Collection in Respiratory Virus Infection Diagnostic Testing. J Clin Microbiol 48: 3340 10.1128/JCM.02235-09 PMC2937679 20610685

[23] DNA Genotek (2012) Omnigene Discover OM-505.

[24] CerTest Biotec Available: http://www.certest.es. Accessed Nov. 12, 2014

[25] Popowitch EB, O'Neill SS, Miller MB (2013) Comparison of the Biofire FilmArray RP, Genmark eSensor RVP, Luminex xTAG RVPv1, and Luminex xTAG RVP fast multiplex assays for detection of respiratory viruses. Journal of clinical microbiology 51: 1528-1533. 10.1128/JCM.03368-12 PMC3647947 23486707

[26] Elliot, A. J., Bermingham, A., Charlett, A., Lackenby, A., Ellis, J., Sadler, C., ... & Zambon, M. (2015). Self-sampling for community respiratory illness: a new tool for national virological surveillance. Euro surveillance: bulletin Européen sur les maladies transmissibles= European communicable disease bulletin, 20(10). 10.2807/1560-7917.es2015.20.10.21058 25788252

[27] Cooper, D. L., Smith, G. E., Chinemana, F., Joseph, C., Loveridge, P., Sebastionpillai, P., ... & Zambon, M. (2008). Linking syndromic surveillance with virological self-sampling. Epidemiology and infection, 136(02), 222-224. 10.1017/S0950268807008412 PMC2870802 17394678

[28] Plymoth, A., Rotzen-Ostlund, M., Zweygberg-Wirgart, B., Sundin, C. G., Ploner, A., Nyren, O., & Linde, A. (2015). Self-sampling for analysis of respiratory viruses in a large-scale epidemiological study in Sweden. Euro surveillance: bulletin Europeen sur les maladies transmissibles= European communicable disease bulletin, 20(11). 10.2807/1560-7917.es2015.20.11.21063 25811646

